# A Novel Role of VEGFC in Cerebral Ischemia With Lung Injury

**DOI:** 10.3389/fnins.2019.00479

**Published:** 2019-05-15

**Authors:** Mu-Dong Wen, Ya Jiang, Jin Huang, Mohammed Al-Hawwas, Qi-Qin Dan, Rui-An Yang, Bing Yuan, Xiao-Ming Zhao, Ling Jiang, Ming-Mei Zhong, Liu-Lin Xiong, Yun-Hui Zhang

**Affiliations:** ^1^Department of Respiration, The First People’s Hospital of Yunnan Province, Kunming, China; ^2^Laboratory Zoology Department, Institute of Neuroscience, Kunming Medical University, Kunming, China; ^3^Department of Anesthesiology, National Traditional Chinese Medicine Clinical Research Base and Western Medicine Translational Medicine Research Center, Affiliated Traditional Chinese Medicine Hospital, Southwest Medical University, Luzhou, China; ^4^Institute of Neurological Disease, Translational Neuroscience Center, West China Hospital, Sichuan University, Chengdu, China; ^5^School of Pharmacy and Medical Sciences, University of South Australia, Adelaide, SA, Australia

**Keywords:** cerebral ischemia, lung injury, VEGFC, protein chip analysis, cell culture

## Abstract

Cerebral ischemia (CI) is a severe brain injury resulting in a variety of motor impairments combined with secondary injury in remote organs, especially the lung. This condition occurs due to insufficient blood supply to the brain during infancy. However, it has a molecular linkage that needs to be thoroughly covered. Here, we report on the role of vascular endothelial growth factor C (VEGFC) in lung injury induced by CI. The middle cerebral artery occlusion (MCAO) was depended to establish the animal model of CI. Rats were used and brain ischemia was confirmed through TTC staining. Serum was used for protein chip analysis to study the proteomic interaction. Immunohistochemistry analyses were used to quantify and locate the VEGFC in the lung and brain. The role of VEGFC was detected by siVEGFC technology in SY5Y, HUCEV, and A549 cell lines, under normal and oxygen glucose deprivation (OGD) conditions *in vitro*. As a result, the TTC staining demonstrated that the model of brain ischemia was successfully established, and MPO experiments reported that lung damage was induced in MCAO rats. VEGFC levels were up-regulated in serum. On the other hand, immunohistochemistry showed that VEGFC increased significantly in the cytoplasm of neurons, the endothelium of small trachea and the lung cells of CI animals. On a functional level, siVEGFC effectively inhibited the proliferation of SY5Y cells and decreased the viability of HUVEC cells in normal cell lines. But under OGD conditions, siVEGFC decreased the growth of HUVEC and increased the viability of A549 cells, while no effect was noticed on SYSY cells. Therefore, we confirmed the different role of VEGFC played in neurons and lung cells in cerebral ischemia-reperfusion injury. These findings may contribute to the understanding the molecular linkage of brain ischemia and lung injury, which therefore provides a new idea for the therapeutic approach to cerebral ischemia-reperfusion.

## Introduction

Cerebral ischemia (CI) is a common neurological disease with high rates of mortality and morbidity (Li et al., 2017; Patel and McMullen, 2017). This condition is triggered by a lack or reduced blood flow to the brain tissue to levels lower than the these required for normal function (Vespa et al., 2005). There are many complications with CI, especially pneumonia that may play a vital role in the death of cerebral ischemia patients which is often overlooked. Similarly, stroke-associated pneumonia (SAP) was firstly reported in 2003. The incidence of SAP was 21% in stroke patients with higher death rates than other patients (Hilker et al., 2003). The annual cost of SAP was about USD 459 million during hospitalization in America (Hannawi et al., 2013). Therefore, it is urgent to explore and understand the mechanism of lung injury induced by CI.

Vascular endothelial growth factor C gene (VEGFC) has been well known to be located on chromosome 4q34 for humans (Janer et al., 2006), and classified with the platelet-derived growth factor/vascular endothelial growth factor (PDGF/VEGF) family (Joukov et al., 1996). As a subtype of VEGF, VEGFC plays a vital role in several biological processes, especially in the endothelium of blood vessels by binding to its receptor, VEGR-3 (Joukov et al., 1996). Recently, VEGFC and CI relation has been established due to a growing amount of evidence of the role of VEGFC in the pathology of CI. During the recovery period of neonatal hypoxia-ischemia (HI), Bain and his coworkers found that VEGFC is briefly induced in the subventricular zone (SVZ), which may relate with the increased blood vessel diameter in SVZ (Bain et al., 2013). By using immunostaining and laser Doppler flowmetry, Bhuiyan et al. (2015) found that VEGF-C/VEGFR-3 signaling is related to hippocampal tolerance to fatal ischemia in ischemic preconditioning (IPC)-induced. Additionally, the observation of decreased VEGFC in chloride intracellular protein 4 (CLIC4) in mice was recently used as an animal model for the disrupted vasculature of multiple organs (Lucitti et al., 2015). Meanwhile, it is known that VEGFC plays an effective role in the development of lymphatic system and pulmonary inflammation (Janer et al., 2006). These effects were linked to the expression of human transcriptional positive cofactor 4 (PC4) in lymph angiogenesis and lymphatic metastasis from lung adenocarcinoma (Tao et al., 2015). Interestingly, ischemia-reperfusion injury (IRI) was cured by VEGF-C/VEGFR3 inhibition, presenting VEGFC inhibitors as a potential strategy for immunomodulatory (Dashkevich et al., 2016). However, the role of VEGFC in CI and its consequent lung injury is yet to be determined. Therefore, we explored the connection of VEGFC and lung injury induced by cerebral ischemia to provide primary evidence in understanding the role of VEGFC for further preclinical application.

## Materials and Methods

### Animals and Grouping

Thirty adult Sprague-Dawley male rats weight 250–300 g were purchased from Department of Zoology of Kunming Medical University. All experiments were carried out in accordance with the principle of the Basel Declaration and recommendation of Animal Experimental Ethical, Kunming Medical University. The protocol was approved by the Animal Experimental Ethical Inspection Committee of Kunming Medical University (reference number: kmmu2018017). They were divided into 2 groups, consisting of 15 rats for each. Middle cerebral artery occlusion (MCAO) was used to establish the cerebral ischemia model for 2 h, which was artificially choked on internal carotid artery by a wire group, then reperfused for 48 h. The other group is designed as sham group (shown in Table 1).

**Table 1:** Animal grouping.

Group	*N*	Treatment	Number	Follow-up
Sham	15	Sham operated	3	IHC
			6	TTC staining
			6	MPO activity; ratios of dry-wet weight, HE staining
			All	Protein microarray
CI/R	15	Lung injury with cerebral Ischemia	3	IHC
			6	TTC staining
			6	MPO activity, ratios of dry-wet weight, HE staining
			All	Protein microarray

### TTC Staining

The cerebral tissues were placed at −80°C for 3–5 min, then sliced into 2 mm coronal sections, and incubated at 37°C in 2% 2,3,5-triphenyltetrazolium chloride (TTC) for 20 min. Subsequently, sections were fixed overnight with 4% paraformaldehyde. In this test, the non-ischemic regions stain red, while the ischemic area become infarct areas and appeared colorless. ImageJ software was used to measure the infarction area of each section. The standard method was described in a previous study, where the infraction volume was expressed as (contralateral hemisphere volume-volume of non-ischemic ipsilateral hemisphere)/the contralateral hemisphere volume (Hu et al., 2009).

### Diagnosis of Lung Injury

After ischemia-reperfusion injury, the lungs were harvested and assayed by determination of pulmonary water content, HE staining and Myeloperoxidase (MPO) estimation with a commercially available kit (Nanjing Jiang Cheng). Briefly, rats were sacrificed 48 h after reperfusion and their lung tissues were collected with 5% homogenate extract and then 0.9 ml of the lysate were mixed with 0.1 ml of the reaction solution at 37°C for 15 min. The color intensity was measured by the absorbance 460 nm with 35 micro plate reader.

### Immunohistochemistry

To detect the VEGFC distribution in brain and lung tissue after MCAO, immunohistochemistry (IHC) staining was performed as previously (Kumar et al., 2017). Brain and lung were fixed in paraffin and cut to 5–20 μm thin sections. The sections were deparaffinized, rehydration, and antigen retrieved with 1% sodium citrate. Then sections were immersed in 3% hydrogen peroxide for 15 min to deactivate endogenous peroxidases, and blocked with 5% goat serum for 10 min at 37°C VEGFC primary antibody (Bioss) was added at 1:400 and incubated overnight at 4 °C. Sections were then washed and incubated with HRP linked secondary antibody followed with DAB staining (MXB, DAB-0031). Finally, hematoxylin solution was used for counterstaining slide drying and observation observed. For immunocytochemical analyses of VEGFC, we randomly selected 5 fields for each section and observed with high-magnification photomicrographs (400×). The expression of VEGFC, were manually quantified by counting the number of varicosities stained with VEGFC antibody staining and the mean of optical density of VEGFC positive neurons in cortex and all positive stained cells in lung which presented as IOD (integrated optical density) over area using Image-Pro Plus 6.0 software (Media Cybernetics, Silver Spring, MD, United States) as previously (Sun et al., 2013).

### Protein Chip

Serum from each group was collected and used in protein chip analysis to find the key proteomics that cause lung injury after cerebral ischemia-reperfusion. The arterial blood of rats was gained from the abdominal aorta from both sham group and ischemia-reperfusion group (*N* = 3). The collected serum was kept at room temperature for 1 h then centrifuged and the supernatant was collected. All samples were sent to KangChen Bio-tech Co., Ltd., in Shanghai, for protein chip detection, followed by routing detection steps provided by KangChen. The concentration of the sample was determined BCA method. Coomassie blue staining was used to check the protein, after electrophoresis gel.

### Protein Quantification Chip Detection and Data Analysis

Antibody chips were blocked in buffer for 30 min and incubated with protein samples at room temperature for 1–2 h. The chips were then rinsed before adding the biotin-labeled antibody, incubated at room temperature for 1–2 h. The chips were washed with washing buffer, and fluorescein-coupled streptavidin was added, then the fluorescence signal was scanned directly using Axon scanners. The protein signals strength was quantified by the fluorescence scanner to obtain the original signal value. Correction was made by subtracting the background values. Differentially expressed proteins were obtained by comparing the values between groups.

### Interference Experiment *in vitro*

Normal and oxygen glucose deprivation (OGD) *in vitro* models were established to detect the function of the VEGFC. Different cell lines were cultured to mimic the cells in the affected organs. SY5Y HUVEC and A549 were used to simulate neurons, pulmonary vascular endothelial cells, and pulmonary epithelial cells, respectively. All cell lines were incubated at 37°C in a 5% CO_2_ incubator, and subcultured when the cells were covered with 80–90% of the plate.

### The Establishment of Interference for VEGFC in Normal Cell Model

Cells were seeded separately in a 6-well culture plate, and 2 mL of complete culture was added to each well in a density allowing them to reach 50% confluence after 1 day. The cells were then transfected with VEGFC siRNA and incubated for 48 h. The model was detected by MTT, Hoechst staining and TUNEL staining.

### The Establishment of Interference for VEGFC in OGD Model

As in a normal model, siRNA was interfered for 48 h. The cell lines were rinsed with sterile PBS before glucose-free DMEM was added for hypoxia and incubated for 1 h in case of SY5Y hypoxia and 4 h for HUVEC and A549. After completion of hypoxia incubation, the sugar-free DMEM were discarded and the complete medium was added for 24 h. MTT, Hoechst staining and TUNEL staining were performed.

### Statistical Analysis

Quantitative variations were assessed by means ± SEM. Data were analyzed via one-way ANOVA with Student’s t assays by SPSS 20.0 after an appropriate correction. The statistical significance (expressed by ^∗^) was defined as *P* < 0.05, and the notable statistical significance (expressed by ^∗∗^) was defined as *P* < 0.01.

## Results

### The Evidence of Cerebral Impairment Indicated by TTC Staining

**FIGURE 1:**
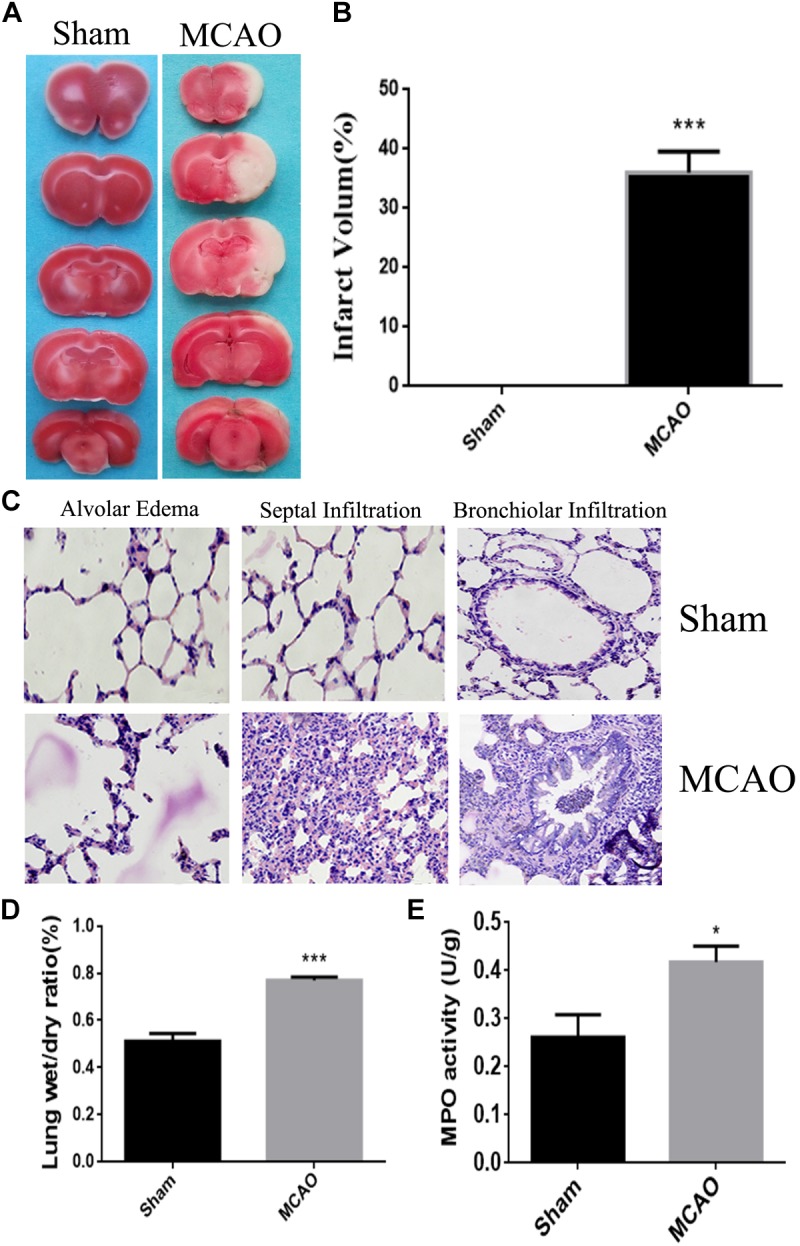
The model of lung injury with cerebral ischemia was successfully constructed. Panel **(A)** means the comparison of TTC staining between sham group and MCAO group, and the white is the ischemic area. Panel **(B)** means the percentage of infarct area. Panel **(C)** means the HE staining of lung tissue edema, seepage and inflammation in sham group and MCAO group. Panel **(D)** means the ratios of dry-wet weight in left lung (the magnification is 400, and the scale is 25 μm). Panel **(E)** means the result of MPO assay. ^∗^*P* < 0.05, ^∗∗∗^*P* < 0.001.

Quantitative analysis of infarct volume after TTC staining showed that the percentage of infarct area in MCAO group was significantly increased, compared with Sham group (*P* < 0.01), which confirmed that cerebral ischemia-reperfusion model has been successfully established (Figure 1A,B).

### Morphological Evidence for Lung Injury From HE Staining

After cerebral ischemia, the lung tissue exhibited as dark red, and was characterized with multiple bleeding points. Under the microscope, the alveolar structure was collapsed, edema was seen in the alveolar septal and the alveolar wall thickened and hemorrhaged. Meanwhile, the lung tissue from Sham group was honeycomb, and the alveolar septum was normal without congestion, edema and significant pathological changes (Figure 1C).

### Changes on the Ratios and Dry-Wet Weight in Lung With Injury

The water percentage in the lung of CI animals was significantly increased (*P* < 0.05) when compared with sham animals. It indicated the edematous condition in the lung of MCAO group (Figure 1D).

### The Result of MPO Assay

The number of neutrophils in the MCAO group was remarkably higher (*P* < 0.05) than those in the control group, which was an obvious sign of inflammation in the lung of ischemic group (Figure 1E).

### The Result of Protein Chip

**FIGURE 2:**
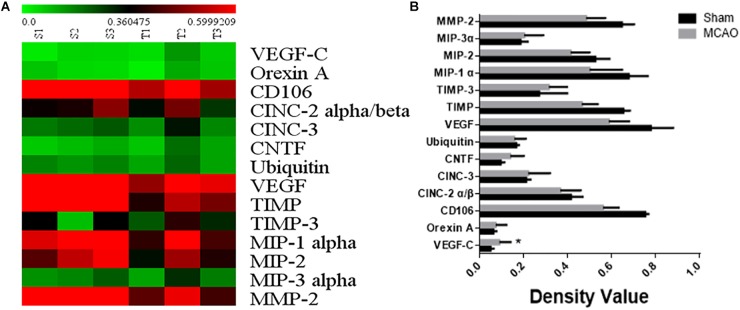
The result of serum protein chip. Panel **(A)** means heat map of serum protein chip. Panel **(B)** means the expressional factor of sham group and MCAO group in protein chip.

The 1.5-fold different proteins were screened by serum protein chip. 14 up-regulated proteins including VEGFC were found out in brain ischemia with lung injury group (Figure 2A,B).

### The Expression and Location of VEGFC in Brain and Lung After MCAO

**FIGURE 3:**
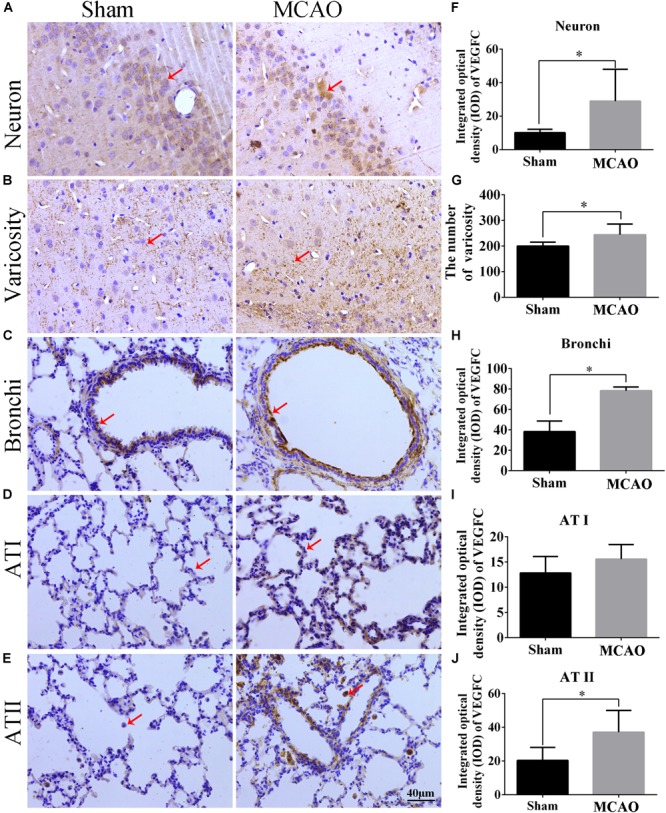
The location and expression of VEGFC in brain and lung after MCAO. Panels **(A–E)** represent the immunohistochemistry after MACO; panel **(A)** means the VEGFC in neurons of cortex (red arrow); panel **(B)** means the VEGFC located in varicosity of cortex (red arrow); panels **(C–E)** mean the location of VEGFC in bronchi, ATI and ATII of lung, respectively (red arrow); panel **(F)** represents the integrated optical density of VEGFC in neurons. Panel **(G)** means the number of varicosity. Panels **(H–J)** mean the integrated optical density of VEGFC in bronchi, ATI and ATII of lung, respectively. The scale bar 40 μm is 400×. ^∗^*P* < 0.05.

Our immunohistochemistry results showed that VEGFC is located in the cytoplasm and varicosity of cortex neurons (Figure 3A,B). Using Sham group as a reference, the levels of VEGFC in neurons markedly increased (*P* = 0.004) in MACO animals (Figure 3F). The number of varicosity of neuron increased significantly in MACO group (*P* = 0.036) (Figure 3G). In the lung, immunohistochemistry showed the VEGFC located in cytoplasm of bronchi, Type I epithelium (AT I), and II epithelium (AT II) (Figure 3C–E). Compared with Sham group, the integrated optical density of VEGFC increased in bronchi (*P* = 0.003), ATI (*P* = 0.337), and ATII (*P* = 0.026) in the MACO group (Figure 3H–J). This indicated that VEGFC regulated blood vessels in the brain released from nerve endings through transmitters, and regulated bronchial contractions in the lungs.

### The Effect of siVEGFC in SY5Y Cells

**FIGURE 4:**
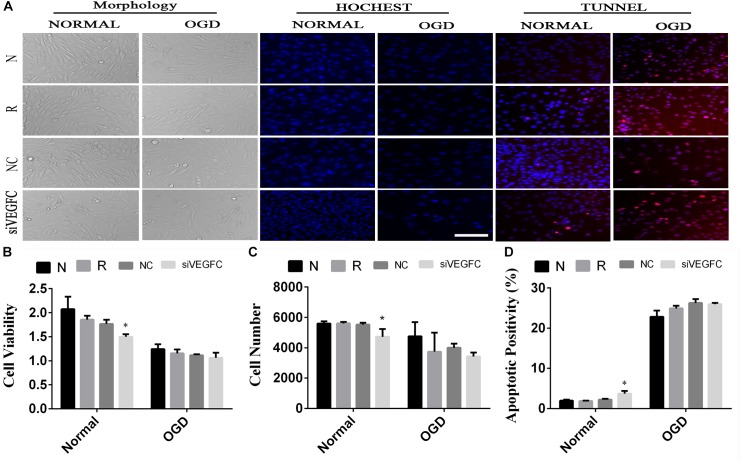
The result of interference assay of SY5Y cells *in vitro*. Panel **(A)** means the cell viability, proliferation and apoptosis of SY5Y in normal and OGD during N, Reagent, NC, and interference of VEGFC (siVEGFC) for SY5Y cells. Panel **(B)** means the histogram of SY5Y’s cell viability during N, reagent, NC, and siVEGFC in normal and OGD. Panel **(C)** means the histogram of SY5Y’s proliferation during N, Reagent, NC, and siVEGFC in normal and OGD. Panel **(D)** means the histogram of SY5Y’s apoptosis during N, Reagent, NC, and siVEGFC in normal and OGD. ^∗^*P* < 0.05.

In order to detect the effect of VEGFC on SY5Y cells, cell viability, cell proliferation and apoptosis were examined by MTT, Hoechst, and TUNEL (Figure 4A). Compared with the NC cell lines, the cell viability in the siVEGFC group decreased remarkably, indicating that siVEGFC interference had affected the cell viability in Normal and OGD condition (Figure 4B). Moreover, the morphological and quantitative changes of SY5Y from Hoechst staining were observed under inverted microscope from day 1 until day 5. Also, the number of cells in the siVEGFC group was decreased, compared with the NC group (Figure 4C). Comparatively, TUNEL staining showed that compared with the NC group, the number of apoptosis of the cells increased in the siVEGFC group, revealing that VEGFC interference increased cell apoptosis (Figure 4D).

### The Effect of siVEGFC in HUVEC Cells

**FIGURE 5:**
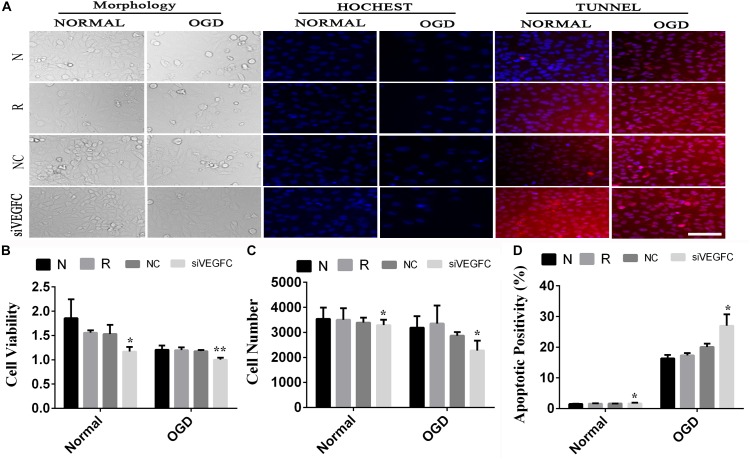
The result of interference assay of HUVEC cells *in vitro*. Panel **(A)** means the cell viability, proliferation, and apoptosis of HUVEC in normal and OGD during N, Reagent, NC, and siVEGFC for HUVEC cells. Panel **(B)** means the histogram of HUVEC’s cell viability during N, Reagent, NC, and siVEGFC in normal and OGD. Panel **(C)** means the histogram of HUVEC’s proliferation during N, Reagent, NC, and siVEGFC in normal and OGD. Panel **(D)** means the histogram of HUVEC’s apoptosis during N, Reagent, NC, and siVEGFC in normal and OGD. ^∗^*P* < 0.05, ^∗∗^*P* < 0.01.

To examine the effect of VEGFC in HUVEC with normal and OGD condition, cell viability, cell proliferation and apoptosis were tested using MTT, Hoechst, and TUNEL. Cell viability in the siVEGFC group was reduced significantly (*P* = 0.027) compared to the NC group. In addition, the number of cells in the siVEGFC interference group (siVEGFC group) was also significantly decreased (*P* = 0.043), while the level of apoptosis was increased in the siVEGFC group. In the OGD group, the effect of siVEGF reported a similar result to the normal one in terms of cell viability. However, the number of cells in the siVEGFC group decreased during ischemia and hypoxia condition compared to the NC group. Cell apoptosis, meanwhile, increased compared with the control group under OGD condition (N + OGD group) (Figure 5).

### The Effect of siVEGFC in A549 Cells

**FIGURE 6:**
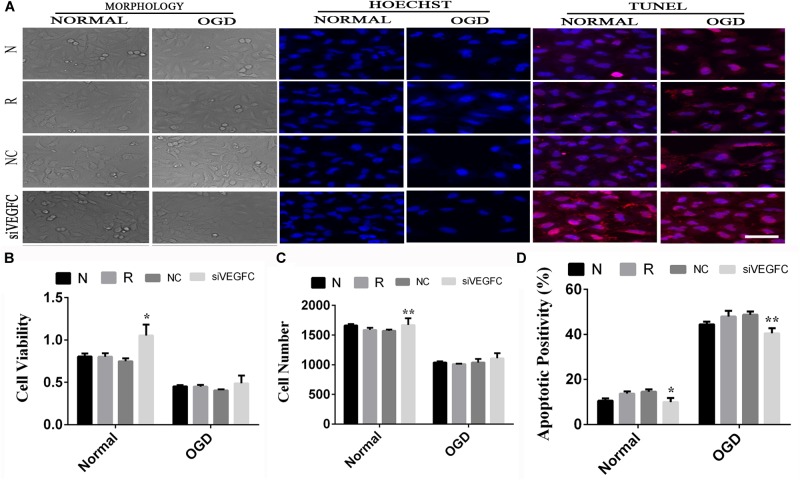
The result of interference assay of A549 cells *in vitro*. Panel **(A)** means the cell viability, proliferation and apoptosis of A549 in normal and OGD during N, Reagent, NC, and siVEGFC for A549 cells. Panel **(B)** means the histogram of A549’s cell viability during N, Reagent, NC, and siVEGFC in normal and OGD. Panel **(C)** means the histogram of A549’s proliferation during N, Reagent, NC, and siVEGFC in normal and OGD. Panel **(D)** means the histogram of A549’s apoptosis during N, Reagent, NC, and siVEGFC in normal and OGD. ^∗^*P* < 0.05, ^∗∗^*P* < 0.01.

We examined the effect of VEGFC in normal A549 cells, through studying cell viability, proliferation and apoptosis. Compared with the negative control group, a significant increase in cell viability was observed, as shown in NC cells after siVEGFC in Figure 6C. The number of cells in the siVEGFC group was significantly increased (*P* = 0.017) while cell apoptosis decreased in comparison to the Normal group (N Group) (*P* = 0.02). In the OGD group, no significant difference was seen in cell viability and proliferation in the siVEGFC group during ischemia and hypoxia compared to the normal group (N group),. However, the amount of cell apoptosis in the siVEGFC group after OGD decreased compared to the normal group (N Group) (Figure 6).

## Discussion

Lung injury, is one of the severe symptom induced by stroke, which can be named as CI associated pneumonia (SAP), in which, it may have two pathway in neurogenic and hematogenic mechanism. We found that the VEGFC increased in lung injury caused by cerebral ischemia, and siVEGFC *in vitro*, confirmed VEGFC is a key protein in lung injury with cerebral ischemia.

### The Establishment of Lung Injury With Cerebral Ischemia

Using the suture method, the ischemia-reperfusion models of rats were successfully established. We found that the lung tissue was dark red and there were bleeding points after cerebral ischemia 2 h, which confirmed that the lung injury can be caused by cerebral ischemia-reperfusion. The Zea-Longa suture method is the most common method for preparing cerebral ischemia models with a constant and exact infarct area. Johnston et al. (1998) confirmed that CI can lead to distant organ damage, which may result from two mechanisms, namely blood and nerves or neurogenic and hematogenic mechanisms. Of these, neurogenic pulmonary edema (NPE) is related the overexcitation of the sympathetic system, which leads to interstitial and interalveolar edema with hemorrhage (Sedy, 2011; Busl and Bleck, 2015). The other mechanism is hematogenic, which is so far unclear. Halladin (2015) found that Ischemia-reperfusion injuries are caused by an excessive production of free radicals or reactive oxygen species. We therefore inferred that those factors can be transported to distant organs and lead to impairment of these other organs. Due to lower resistance and greater blood volume, the lung became one of the most impaired organs with a higher death rate (Rincon et al., 2014).

### The Implication of VEGFC Increase in Serum From Protein Chip

In this study, we used the protein chip of serum to find differential proteins that cause lung injury after cerebral ischemia-reperfusion. The protein chip is a high-throughput method for tracking the interaction and activity of proteins, and massively determines their function (Melton, 2004). There are numerous advantages to the protein chip, such as high sensitivity, economy, speed and so on (Mitchell, 2002). High-throughput technology has been well developed for protein analysis because it followed DNA microarray development (Hall et al., 2007). In this study, VEGFC was found as an over-expression protein in the model of lung injury induced by cerebral ischemia, which has not been reported in recent literature.

### The Implication of VEGFC Expression in the Brain and Lung

In this study, we found extensive positive stains distributed in the cortex, specifically in neurons and their neuritis (marked as varicosity). This suggests that VEGFC is imported in maintenance neuronal function and neuro-transduction. Previous literature showed that VEGF, as a growth factor, could be expressed in both neurons and endothelium in blood vessels (Joukov et al., 1996). Our study was the first to show that VEGFC was expressed in neurons. Importantly, several processes extended from neurons exhibited VEGFC positive staining, and a multitude of extension node in the process could be seen, suggesting that VEGFC is distributed in varicosity. This indicated that VEGFC has some functions that involve synaptic information transduction. This is a very important finding inour study, which is not similarly reported in past literature. In the lung, VEGFC positive was found both in epithelia of the lung and small trachea, which suggests that VEGFC is involved in physiological and pathological function. This is also a new presentation for VEGFC in epithelia of the lung. As to the function of VEGFC in the brain and lung, it is not clear from the evidence. The up-regulation of VEGFC in neurons and lung cells suggested that VEGFC has been involved in the process of brain ischemia with lung injury. As a trophic factor, VEGFC may be useful in the self-recovery in injured brains and lungs, despite the fact that the mechanism of this self-recovery may be weak. Our experiment provided novel evidence to address a new role of VEGFC in lung injury induced by CI.

### The Different Role of VEGFC From Interference Assay *in vitro* by Using siRNA

We used SY5Y cell lines to simulate neurons, HUVEC cell lines to simulate pulmonary vascular endothelial cells, and A549 cell lines to simulate pulmonary epithelial cells, to understand the role of VEGFC in our study. The siRNA was then used to interfere the three kinds of cells. Small interfering RNA (siRNA), double-stranded RNA molecules with 20–25 base pairs in length, play a vital role in interference technology via degrading mRNA with complementary nucleotide sequences, and resulted in no translation (Xia et al., 2002; Agrawal et al., 2003). In this study, siVEGFC inhibited the proliferation of SY5Y cells, the viability of HUVEC cells, and apoptosis while producing a reversed result in A549 cell. In OGD condition, siVEGFC inhibited the apoptosis of A549 cells, but increased the apoptosis of HUVEC, while there was no effect for SY5Y. It appears that the cerebral tissue may express more VEGFC to protect itself during the ischemia, but it is harmful in lung tissue. When reperfusion is coming, the VEGFC in the lung may cause the lung injury. Guo et al. (2013) found that SiRNA can block endogenous VEGFC gene and protein expression in gastric cancer cells. Yao et al. (2013) found that VEGFC-shRNA can effectively inhibit the migration of gastric cancer cells *in vivo* and delay the tumorigenicity and lymph angiogenesis in nude mice. In addition, VEGFC may participate in the glial reaction through autocrine or paracrine mechanisms in cerebral ischemia and it plays a vital role in adult hippocampal neurogenesis during ischemic injury (Shin et al., 2008). El-Chemaly S found that airway epithelial and inflammatory cells expressed VEGFC (El-Chemaly et al., 2008), while TNF-α could up-regulate the expression of VEGFC to promote the lymph angiogenesis of GBC by binding to NF-κB and VEGFC (Du et al., 2014). Therefore, the up-regulation of VEGFC in lungs may be harmful, and siVEGFC may provide a useful method to prevent lung injury.

### The Possible Mechanism for VEGFC in Brain Ischemia Inducing Lung Injury

The main function of VEGFC is lymph-angiogenesis, by binding its receptor VEGFR-3, VEGFR-3 to promote survival, growth and migration, and working on lymphatic endothelial cells (Alitalo and Carmeliet, 2002). It also promotes vascular growth and regulates its permeability, and the role of blood vessels can be mediated by its main receptor VEGFR-3 or its secondary receptor VEGFR-2 (Tammela et al., 2008). VEGFC is also important for neurodevelopmental and blood pressure regulation (Le Bras et al., 2006; Machnik et al., 2009). Wang X found that VEGFC plays a vital role in the pathogenesis for inflammatory bowel diseases (IBD), which aggravated intestinal inflammation through stimulation (Wang et al., 2016). The overexpression of VEGFC leads to hyperplastic lymphatic vessels, which do not enhance lymphatic drainage, but do promote the delivery of phagocytic cells to draining lymph nodes (Papadakou et al., 2017). Therefore, VEGFC may regulate blood vessels in the brain through transmitters released from nerve endings for bronchial contractions, and regulating cell function in the lungs.

**FIGURE 7:**
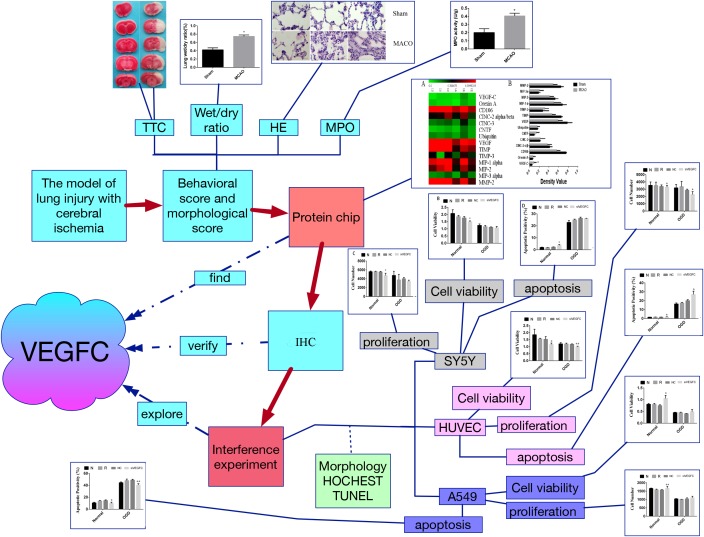
The flow chart of this study. Firstly, the model of lung injury of cerebral ischemia was established. Secondly, the morphological score were evaluated by TTC staining, wet/dry ratio, HE staining and MPO assay, which verify that the cerebral ischemia can cause the lung injury. Third, the protein chip was used to find the key factor, namely VEGFC. Fourthly, the VEGFC was detected by Q-PCR. Finally, we carried out the interference experiment by siVEGFC on the SY5Y cells, HUVEC cells and A549 cells, and the cell viability proliferation and apoptosis was observed via morphology staining, HOCHEST staining and TUNEL staining. ^∗^*P* < 0.05, ^∗∗^*P* < 0.01.

In summary, by using the model of lung injury with cerebral ischemia, we found that VEGFC is useful for neurons in the brain to protect itself during the ischemia, but that it is harmful in lung tissue. It may offer a novel idea to treat lung injury by targeting VEGFC after cerebral ischemia-reperfusion (Figure 7).

## Author Contributions

Y-HZ and L-LX participated in the guidance of the study and revision of the manuscript. M-DW, YJ, and JH were responsible for the manuscript writing and revising, data description and manuscript submission. Q-QD established the model, performed the TTC staining, and harvested the tissue. MA-H participated in data analysis and writing of the manuscript. R-AY and Q-QD analyzed the data about protein chip. BY and X-MZ transfected the virus and performed the MTT method. LJ and M-MZ performed the PCR experiments. All authors read and approved the final version of the manuscript.

## Conflict of Interest Statement

The authors declare that the research was conducted in the absence of any commercial or financial relationships that could be construed as a potential conflict of interest.
